# SWATH^HM^-Based Metabolomics of Follicular Fluid in Patients Shows That Progesterone Adversely Affects Oocyte Quality

**DOI:** 10.1155/2018/1780391

**Published:** 2018-05-22

**Authors:** Zhengao Sun, Jingyan Song, Xingxing Zhang, Aijuan Wang, Ying Guo, Yi Yang, Xiaoming Wang, Kaiyue Xu, Jifeng Deng

**Affiliations:** ^1^Reproductive and Genetic Center of Integrated Traditional and Western Medicine, The Affiliated Hospital of Shandong University of Traditional Chinese Medicine, Jinan 250011, China; ^2^Department of Gynecology and Obstetrics of Traditional Chinese Medicine, The First Clinical College, Shandong University of Traditional Chinese Medicine, Jinan 250014, China; ^3^School of Bioscience & Bioengineering, South China University of Technology, Guangzhou 510640, China

## Abstract

**Objective:**

We reveal the relationship between progesterone level in follicular fluid and oocyte quality based on sequential window acquisition of all theoretical fragment-ion spectra (SWATH™), a powerful high-resolution mass spectrometric data independent acquisition technique.

**Method:**

Follicular fluid samples were collected from 22 subjects (the level of progesterone > 1.5 ng/mL) of progesterone group, as well as from 22 subjects (the level of progesterone < 1.5 ng/mL) of control group, and analyzed using UPLC-Q-TOF. All methods were performed in accordance with ISO 9001:2008. Novel SWATH acquisition mode on an ultra-high performance liquid chromatography coupled with hybrid triple quadrupole time-of-flight mass spectrometry (with resolving power 20,000–40,000) was investigated for the analysis of human follicular fluid. The principal component variable grouping detects intersample variable correlation and groups variables with similar profiles which simplifies interpretation and highlights related ions and fragments. It can also extract product ion spectra from the data collected by fragmenting a wide precursor ion window.

**Results:**

Follicular fluid from the two groups differed with respect to five metabolites. Follicular fluid from the progesterone group contained elevated levels of 8-hydroxyguanosine and 4-hydroxynonenal and reduced levels of ATP, estradiol, and L-carnitine. The increased progesterone level on the day of HCG injection could negatively impact oocyte quality, thus reducing the pregnancy rate of IVF patients.

## 1. Introduction

At present,* in vitro* fertilization (IVF) and intracytoplasmic sperm injection are the main treatments available for infertility. High egg quality and good endometrial receptivity are key conditions for the success of these procedures. Controlled ovarian hyperstimulation is an important part of IVF and intracytoplasmic sperm injection technology [1]. Gonadotropin-releasing hormone can induce the production and maturation of multiple eggs, resulting in an increase in pregnancy rates [2]. Simultaneously, various endocrine changes arise due to the development of multiple follicles and* corpora lutea*. Embryo quality and endometrial receptivity may also be influenced by the exposure to a high level of steroids in the internal environment, which may affect the outcome of pregnancy [3]. During IVF treatment, the serum level of progesterone commonly increases on the day of human chorionic gonadotropin (HCG) administration [4]. Whether the level of progesterone in the serum on the day of HCG administration affects oocyte quality is a controversial topic [5, 6]. Follicular fluid provides an ideal microenvironment for oocyte development, containing the nutrients required for follicle growth and maturation. The presence of altered metabolic components in follicular fluid has been related to follicle quality and developmental potential.

Metabolomics, which is the identification and quantification of all metabolites in biological systems, can be used to find potential biomarkers for studying the relative biochemical pathways in follicular fluid. Although high-resolution mass spectrometry (HRMS) such as time-of-flight (TOF) has become a conventional tool for metabolomics study [7, 8], identification of low level differential endogenous metabolites is still very difficult. The *m*/*z* (mass-to-charge ratio) of the precursor and product ions was recorded in MS and MS/MS spectra to provide crucial information for the elemental composition analysis and structure elucidation. The hit rates of the MS/MS spectrum played a decisive role in differential endogenous metabolites identification. Compared to the traditional information dependent acquisition (IDA) method [9, 10], SWATH significantly improved the hit rate of low level endogenous metabolites because it could sequentially obtain all MS/MS spectra of all mass windows across the specified mass range [11]. Moreover, MS^E^ or MS^ALL^ technique has no selection of precursor ions to trigger the acquisition of fragment-ion spectra [12]. Novel SWATH technique has higher selectivity and sensitivity than MS^E^ or MS^ALL^ [13, 14]. Another advantage of SWATH acquisition resides in the possibility of reprocessing the same data set to obtain previously unidentified features without reacquiring the sample [15].

In this study, we retrospectively analyzed 44 follicular fluid samples, collected during IVF cycles. To the best of our knowledge, the work presented here is the first application of the SWATH technique for investigating the relationship between the levels of serum progesterone on the day of HCG administration and oocyte quality, as well as its impact on the outcome of pregnancy.

## 2. Clinical Characteristics of Patients

All subjects (*n* = 62) were recruited from the Integrative Medicine Research Centre of Reproduction and Heredity, of the Affiliated Hospital of Shandong University of Traditional Chinese Medicine, from January to December in 2015. The study was approved by the Health Authorities and Ethics Committees of the Affiliated Hospital of Shandong University of Traditional Chinese Medicine. All study participants signed an informed consent form before the start of the study. Study participants were divided into a progesterone group (the level of progesterone > 1.5) and a control group (the level of progesterone < 1.5). Their clinical background was listed in Table 1. The mean BMI of the subjects was a little less than 18.5 according to WHO rules because most of women in China pursue a thin body. It had no effect on oocyte and pregnancy rates from our clinical experience. All participants had a normal menstrual cycle, without clinical or biochemical hyperandrogenism. The age of the participants varied between 24 and 46 years. During the selection process, the following exclusion criteria were used: (1) patients with severe mental diseases, acute urogenital inflammation, or sexually transmitted diseases; (2) patients who received hormonal therapy in the latest 3 months; (3) patients with hereditary diseases that are incompatible with pregnancy; (4) drug abusers or those having harmful addiction; and (5) patients in touch with radiation, toxins, or drugs that could cause malformations. 18 women were excluded according to the above exclusion criteria.

Prior to entering the trial, 44 women signed informed consents. On the basis of established protocols, all patients underwent controlled ovarian hyperstimulation (COH). When the size of at least three follicles reached more than 18 mm, 10,000 IU human chorionic gonadotropin (hCG) (Choriomon, IBSA, Switzerland) was administered intramuscularly, 34–38 h after hCG injection under ultrasound guidance. Follicles larger than 18 mm in diameter were aspirated using a 17-gauge Cook needle. Subsequently, oocytes were retrieved [16–18]. After oocyte isolation, follicular fluid from 3 mature follicles (larger than 18 mm) was pooled and centrifuged at 14,000 ×g for 20 min, to remove cells and insoluble particles. The supernatant was transferred to sterile cryovials and stored at −80°C for further study. Specimens with blood contamination were discarded [19]. All operations were performed in accordance with ISO 9001:2008.

## 3. Sample Preparation

Follicular fluid samples of 100 *μ*L were mixed with 300 *μ*L of methanol containing 4 *μ*M of gemfibrozil (purity: >98%; Sigma; Louis, MO, USA) and isotope-labeled d3-palmitic acid (purity: >98%; Sigma; Louis, MO, USA). The mixture was vortexed for 5 min and then centrifuged at 14000 ×g for 30 min, at 4°C. The supernatant was then transferred to an autosampler plate for analysis.

## 4. Method Condition

Aliquots of 2 *μ*L supernatant were injected into the ultra-performance liquid chromatography tandem Triple TOF 5600 system (AB SCIEX, CA, USA) in random order, to avoid complications caused by artifacts related to injection order and occasional changes in instrumental efficiency. The liquid chromatography system consisted of a reverse-phase 2.1*∗*100 mm ACQUITY UPLC® BEH C_18_ 1.7 *μ*m column (Waters Corp., USA), with a gradient mobile phase composed of 0.1% formic acid solution (A) and acetonitrile containing 0.1% formic acid solution (B). The gradient was kept at 95% A for 1 min, increased to 100% B over the next 6 min, and then returned to 95% A from 9 min to 9.2 min. The total run time was 12 min. Nitrogen was used as a nebulizer (60 L/h) and desolvation gas (60 L/h). Source temperature was set at 550°C. The declustering potential and collision energy were set at 5500 and 25 V in positive mode (−4500 and −25 V in negative mode), respectively. Data was acquired in both positive and negative modes on a Q-TOF mass-spectrophotometer, using Analyst 1.7.1 TF software, operated in full-scan mode and in product ion scan mode at *m*/*z* 100–1200.

## 5. Reproducibility and Accuracy

Eleven quality control (QC) samples (one QC after each four follicular fluid samples) were prepared by mixing equal volumes of different individual follicular fluid samples. The reproducibility of the main background ions and the internal standard ions in QC samples, such as midazolam (*m*/*z* 326.0860), was accessed the reproducibility and the reliability of the UPLC-MS system using the relative standard deviation (RSD). The internal standard ion (*m*/*z* 326.0860) was detected for assessing the accuracy of the method via comparisons with the theoretical mass.

## 6. Data Analysis

In total, 44 follicular fluid samples were analyzed via ultra-performance liquid chromatography coupled to time-of-flight mass spectrometry (UPLC-TOF-MS) using SWATH mode. Data was processed using PeakView software (AB SCIEX, CA, USA) for qualitative analyses and MarkerView software (AB SCIEX, CA, USA) for Multivariate Analysis (MVA). In large-scale nontargeted liquid chromatography mass spectrometry (LC-MS) metabolomic measurements, the reproducibility may be influenced by source contamination or the maintenance and cleaning of the mass spectrometer. Normalization is a common preprocessing method to decrease systematic change. All ions were extracted and aligned using the MarkerView software (AB SCIEX, CA, USA), to generate a data matrix consisting of peak areas corresponding to a unique *m*/*z* and retention time (RT), with normalization. After aligning peaks, the zero-values were removed using the modified 80% rule. The software was also used to generate a score plot and a loading plot by principal component analysis. The loading plot was used to define ions exerting a major influence on the group membership. The list of contributing ions was produced based on *P* values below 0.05. The list of contributors was further investigated to identify candidate biomarkers in the progesterone group, by comparison with the control group. The predictive ability of the model was assessed by internal validation, using the 7-fold cross-validation and response permutation testing. All data were presented as mean ± standard deviation and were displayed using GraphPad Prism. Student's *t*-test was used for statistical comparisons. *P* values of less than 0.05 were considered indicative of statistically significant differences. High contribution score, accurate mass, isotope patterns, and mass spectrometric fragmentation patterns were used to search databases for identifying the differential ions, including KEGG, PubChem compound, METLIN, Madison Metabolomics Consortium Database, and the Human Database.

## 7. Results

Eleven QC samples were used to assess the reproducibility and the reliability of the UPLC-MS system. The QC samples were tightly clustered together via PCA analysis. Moreover, the RSD of internal standard ion, such as midazolam (*m*/*z* 326.0860), was less than 11.3% among the QC samples. It indicates that the reproducibility and the reliability of the UPLC-MS system are good. The internal standard ion (*m*/*z* 326.0860) was detected at *m*/*z* 326.0866. This was less than 1 mDa via comparisons with the theoretical mass. This showed that the accuracy of the method was adequate for detecting the unknown samples. Chromatography residue was also assessed using the internal standard peak area in blank sample following follicular fluid sample. Internal standard peak was not detected in blank sample, which indicates that there is no chromatography residue. These indicated that the analytical method was adequate for use in the metabolomics study.

**Figure 1:**
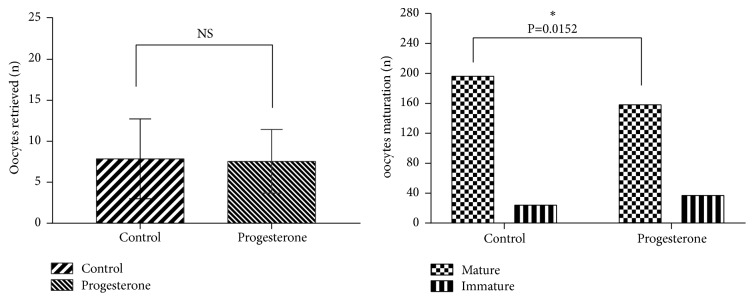
Oocytes retrieved and mature rate.

**Figure 2:**
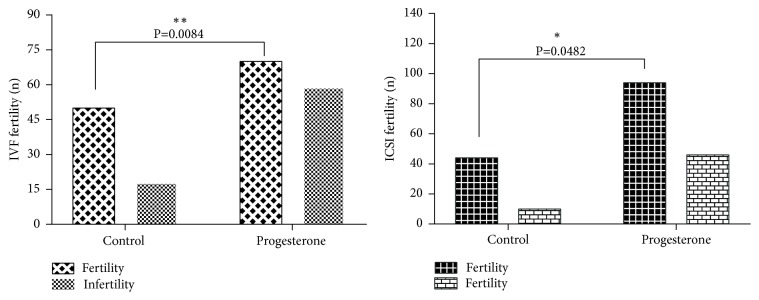
IVF fertility rate and ICSI fertility rate.

The oocytes retrieved and mature rate of 44 subjects from progesterone group and control group were shown in Figure 1 (oocytes retrieved and mature rate). There are no differences in the number of oocytes retrieved between these two groups. The oocyte quality is mainly evaluated by the maturity of oocyte. The mature rate of oocytes of progesterone group was lower than that of control group. The ICSI fertility rate was also significantly low in progesterone group from Figure 2 (IVF fertility rate and ICSI fertility rate).

**Figure 3:**
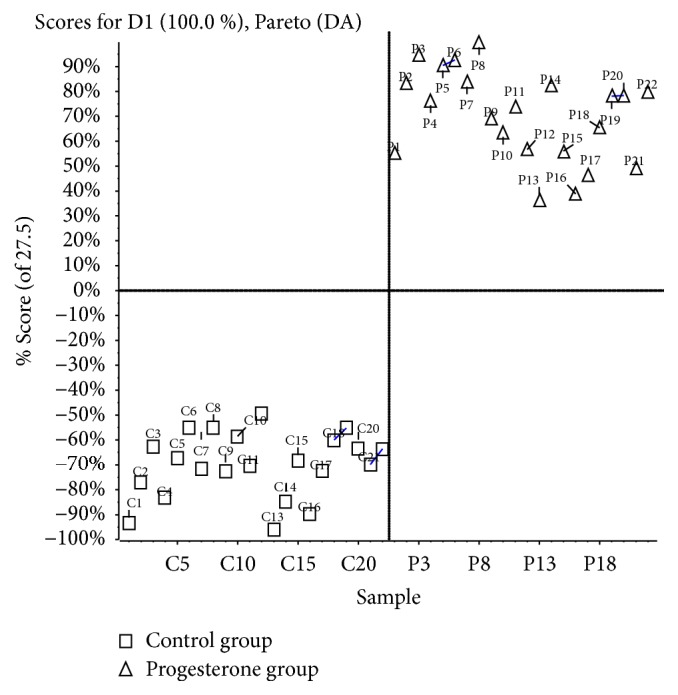
Score plots of PCA analysis for all metabolites in follicular fluid samples.

The progesterone group was completely separated from control group, as shown in Figure 3 (score plot). It suggested that some metabolites were significantly changed in the follicular fluid due to the increased level of progesterone. The list of contributing metabolites was produced based on obtained *P* values below 0.05. The metabolites were validated with accurate mass, isotope patterns, and mass spectrometric fragmentation patterns.

**Figure 4:**
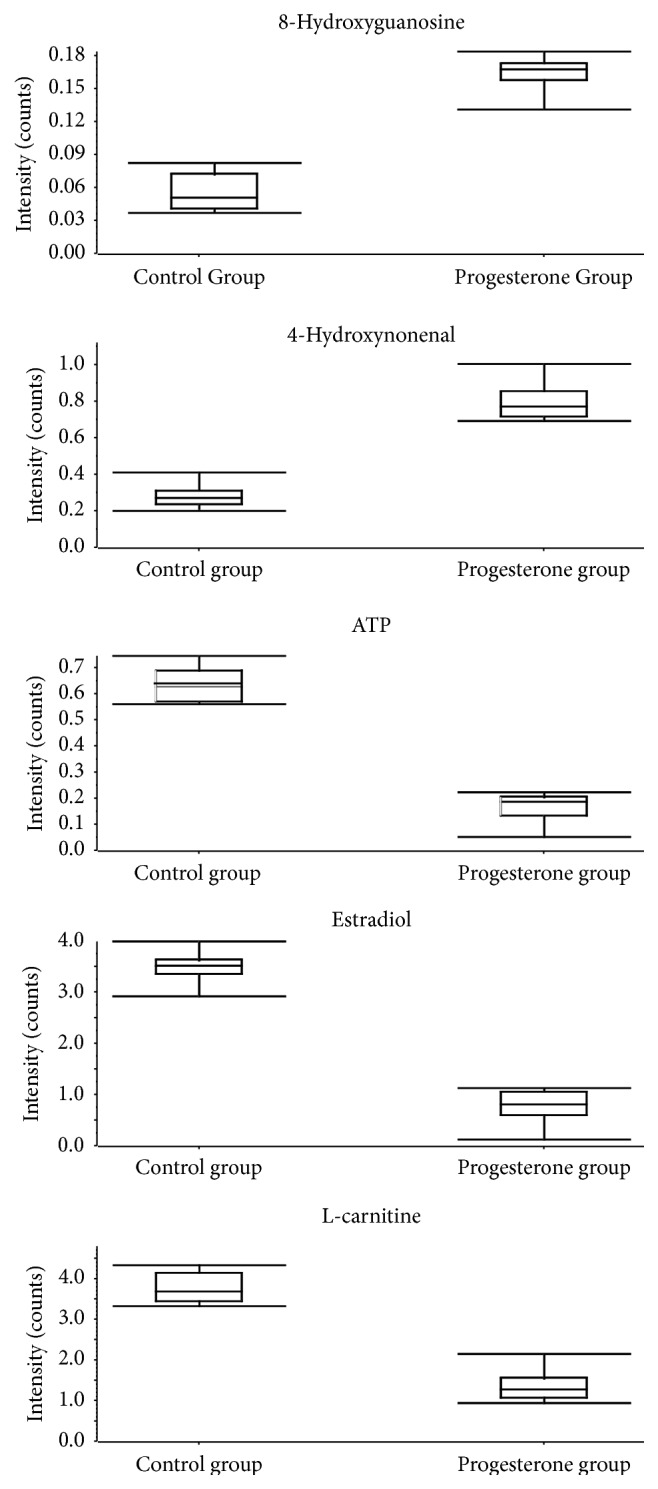
Metabolite profiles of the five metabolites obtained from the quantitative analysis of the subjects.

The differential metabolite M1 showed the [M+H]^+^ ion at *m*/*z* 284.0979. The elution time of M1 was 3.23 min in the UPLC chromatogram. Its molecular formula was inferenced as C_10_H_13_N_5_O_5_ according to its accurate mass and isotope patterns. A series of characteristic products ions at *m*/*z* 266.0832, 168.0511, 140.0575, 123.0304, and 112.0621 by successive loss of H_2_O, C_5_H_8_O_3_, C_6_H_8_O_4_, C_6_H_11_O_4_N, and C_7_H_8_O_5_ were observed. The structure of M1 was inferenced as 8-hydroxyguanosine based on the above MS and MS2 information. The differential metabolite M2 showed the [M-H]^−^ ion at *m*/*z* 155.1081. The elution time of M2 was 1.65 min in the UPLC chromatogram. Its molecular formula was inferenced as C_9_H_16_O_2_ according to its accurate mass and isotope patterns. A series of characteristic products ions at *m*/*z* 137.0964 and 85.0275 by successive loss of H_2_O and C_5_H_10_ were observed. The structure of M2 was inferenced as 4-hydroxynonenal based on the above MS and MS2 information. The differential metabolite M3 showed the [M+H]^+^ ion at *m*/*z* 508.0042. The elution time of M3 was 6.88 min in the UPLC chromatogram. Its molecular formula was inferenced as C_10_H_16_N_5_O_13_P_3_ according to its accurate mass and isotope patterns. A series of characteristic products ions at *m*/*z* 250.0917 and 135.0526 by successive loss of H_5_O_10_P_3_ and C_5_H_12_O_13_P_3_ were observed. The structure of M3 was inferenced as ATP based on the above MS and MS2 information. The differential metabolite M4 showed the [M−H]^−^ ion at *m*/*z* 271.1694. The elution time of M4 was 9.28 min in the UPLC chromatogram. Its molecular formula was inferenced as C_18_H_24_NO_2_ according to its accurate mass and isotope patterns. A series of characteristic products ions at *m*/*z* 183.0810 and 145.0685 by successive loss of C_5_H_12_O and C_8_H_14_O were observed. The structure of M4 was inferenced as estradiol based on the above MS and MS2 information. The differential metabolite M5 showed the [M+H]^+^ ion at *m*/*z* 162.1114. The elution time of M5 was 1.68 min in the UPLC chromatogram. Its molecular formula was inferenced as C_7_H_15_NO_3_ according to its accurate mass and isotope patterns. A series of characteristic products ions at *m*/*z* 103.0391, 85.0309, and 57.0362 by successive loss of C_3_H_9_N, C_3_H_11_NO, and C_4_H_11_NO_2_ were observed. The structure of M5 was inferenced as L-carnitine based on the above MS and MS2 information. The results were shown in Table 2. As seen in Figure 4 (metabolite profiles), the differences of the 5 metabolites between the progesterone group and the control group were displayed with the Graph Pad Prism.

## 8. Discussion

SWATH is a new online data acquisition method for the independent parameter acquisition of compounds. SWATH method could allow for the detection of all peaks and acquire the corresponding MS/MS spectrum while not requiring the setting of mass defect filters prior to injection. The instrument can rapidly and sequentially acquire the MS/MS of all mass windows across the specified mass range. Therefore, compared to IDA, SWATH can obviously improve the hit rates of MS/MS spectrum. At the same time, the quality of the MS2 spectrum using SWATH method could be greatly improved compared with MS^E^ or MS^ALL^. In the study, the differential metabolites between two groups were obtained by the SWATH method. Based on the above advantages, SWATH is well suited for experimental applications of nontarget experimental objectives.

During IVF treatment, serum progesterone level commonly increases on the day of HCG injection. However, whether the elevated serum progesterone level affects oocyte quality has not reached consensus among researchers. Some researchers believe that the decreased clinical pregnancy rate is likely due to the decreased endometrial receptivity, rather than the low quality of eggs and embryos [5], because the increased progesterone level causes the endometrial implantation window to open early. In fact, the pregnancy rate was affected by many factors such as age, polycystic ovary syndrome, endometriosis, and oocyte quality. In our study, the impact of the elevated serum progesterone levels on oocyte quality is discussed.

It is impossible for all researchers to directly use human oocytes for experiments, due to ethical reasons. Follicular fluid, cumulus and granulosa cells, and peritoneal fluid are often used to investigate the quality and developmental potential of oocytes, as they are closely related to oocyte production and maturation. Moreover, oxidative stress, abnormal energy metabolism, abnormal hormone metabolism, and increased inflammatory responses can all negatively impact oocyte quality.

Botero-Ruiz et al. found that the cleavage and pregnancy rates vary directly with the amount of estradiol in the follicles [20]. Estradiol is a marker of oocyte maturation and an indicator of oocyte quality. In our study, the estradiol levels in the progesterone group were reduced, which suggests that the elevated progesterone levels caused abnormal hormone metabolism and resulted in a decline in oocyte quality.

Oxidative stress is associated with important pathophysiological events in many diseases. The production of free radicals and lipid peroxidation products may be an important factor for oocyte quality during follicular growth and ovulation. 8-Hydroxyguanosine (8-OHdG) and 4-hydroxynonenal (4HNE) are the most common markers for evaluating oxidative stress [21, 22]. These oxidation products elicit a variety of pathological effects, such as enzyme inhibition, inhibition of DNA and RNA synthesis, inhibition of protein synthesis, and the induction of heat shock proteins. In our study, 8-OHdG and 4HNE levels increased significantly when the progesterone level increased in the IVF cycle, indicating that higher progesterone causes more oxidative stress.

Mitochondria, as the energy factory of the cells, can produce large amounts of ATP to provide energy for cellular activities, by aerobic respiration. It participates in the process of steroidogenesis and cell senescence. Xu et al. found that the number of abnormal mitochondria in particle cells increased significantly in patients with wild endometriosis [23]. Oocyte aging is mainly caused by mitochondrial dysfunction due to the declined oxidative phosphorylation and decreased ATP production [24]. Mitochondrial dysfunction can lead to oocyte spindle defects and chromosomal disorders which affect oocyte development, maturation, and fertilization. In our study, ATP levels were reduced in the oocytes of patients in the progesterone group. This suggests that the quality of oocytes was affected by the abnormal energy metabolism caused by the elevated progesterone levels.

In previous studies, L-carnitine could protect oocytes and embryos from sustained damage, improving the oocyte quality and embryonic development potential, as well as the pregnancy rate [25, 26]. In our study, the level of L-carnitine was decreased in the progesterone group compared to the control group. This also indicates that the elevated progesterone level can affect oocyte quality. The fertilization and/or pregnancy outcome was significantly different between the progesterone group and the control group. The high level of progesterone was one important factor for low fertilization rate due to poor oocyte quality.

## 9. Conclusions

At present, the effect of progesterone levels on the quality of oocytes is still controversial. The mechanism behind the impact of progesterone level on oocyte quality is a topic of debate and active research in the field of reproductive medicine. To our knowledge, no studies have been published using a metabolomics approach to analyze the human follicular fluid based on SWATH technique. Novel online SWATH technique was the first time used to investigate the relationship of level of progesterone with oocyte quality. Higher progesterone level compromises oocyte quality, as reflected in key metabolite levels. For example, the estradiol levels in the progesterone group were reduced, which suggests that the elevated progesterone levels caused abnormal hormone metabolism and resulted in a decline in oocyte quality.

## Figures and Tables

**Table 1:** The clinical background of progesterone group (*P* > 1.5 ng/mL) and control group (*P* < 1.5 ng/mL).

Group	Subjects	Age (years)	FSH (U/L)	BMI (kg/m^2^)
Control	22	31.5 ± 6.8	6.8 ± 1.6	18.3 ± 1.2
Progesterone	22	30.7 ± 7.6	7.5 ± 1.0	17.9 ± 1.4

**Table 2:** Characterization of the differential metabolites between progesterone group (*P* > 1.5 ng/mL) and control group (*P* < 1.5 ng/mL) in follicular fluid.

*T*_*R*_ (min)	*m*/*z*	Molecular Formula	Identity (Progesterone group vs Control group)	Error (mDa)	Fold change (P/C)	*T*-test (*P*)
3.23	284.0979	C_10_H_13_N_5_O_5_	8-Hydroxyguanosine	−1.0	3.7	<0.01
1.65	155.1081	C_9_H_16_O_2_	4-Hydroxynonenal	1.4	2.5	<0.01
6.88	508.0042	C_10_H_16_N_5_O_13_P_3_	ATP	0.6	0.24	<0.05
9.28	271.1689	C_18_H_24_O_2_	Estradiol	−0.4	0.19	<0.01
1.68	162.1118	C_7_H_15_NO_3_	L-carnitine	−0.7	0.33	<0.01

## References

[1] Bosch E., Labarta E., Crespo J. (2010). Circulating progesterone levels and ongoing pregnancy rates in controlled ovarian stimulation cycles for in vitro fertilization: Analysis of over 4000 cycles. *Human Reproduction*.

[2] Huang R., Fang C., Xu S., Yi Y., Liang X. (2012). Premature progesterone rise negatively correlated with live birth rate in IVF cycles with GnRH agonist: An analysis of 2,566 cycles. *Fertility and Sterility*.

[3] Wu Z., Li R., Ma Y. (2012). Effect of HCG-day serum progesterone and oestradiol concentrations on pregnancy outcomes in GnRH agonist cycles. *Reproductive BioMedicine Online*.

[4] Al-Azemi M., Kyrou D., Kolibianakis E. M. (2012). Elevated progesterone during ovarian stimulation for IVF. *Reproductive BioMedicine Online*.

[5] Lahoud R., Kwik M., Ryan J., Al-Jefout M., Foley J., Illingworth P. (2012). Elevated progesterone in GnRH agonist down regulated in vitro fertilisation (IVFICSI) cycles reduces live birth rates but not embryo quality. *Archives of Gynecology and Obstetrics*.

[6] Xu B., Li Z., Zhang H. (2012). Serum progesterone level effects on the outcome of in vitro fertilization in patients with different ovarian response: an analysis of more than 10,000 cycles. *Fertility and Sterility*.

[7] Yao D. G., Li Z., Huo C. H. (2016). Identification of in vitro and in vivo, metabolites of alantolactone by UPLC-TOF-MS/MS. *Journal of Chromatography B*.

[8] Qiang R., Wang Y. L., Wang M. L. (2016). Screening and identification of themetabolites in rat urine and feces after oral administration of Lycopus lucidus,Turcz extract by UHPLC-Q-TOF-MS mass spectrometry. *Journal of Chromatography B*.

[9] Sun Y. P., Jia P. P., Yuan L. (2015). Investigating the in vitro, stereoselective metabolismof m-nisoldipine enantiomers: characterization of metabolites andcytochrome P450 isoforms involved. *Biomedical Chromatography*.

[10] Lin M. Y., Zhao S. H., Wang Z. Q. (2014). Identification of metabolites ofdeoxyschizandrin in rats by UPLC-Q-TOF-MS/MS based on multiple massdefect filter data acquisition and multiple data processing techniques. *Journal of Chromatography B*.

[11] Xie W., Jin Y., Hou L. (2017). A practical strategy for the characterization of ponicidin metabolites in vivo and in vitro by UHPLC-Q-TOF-MS based on nontargeted SWATH data acquisition. *Journal of Pharmaceutical and Biomedical Analysis*.

[12] Bilbao A., Varesio E., Luban J. (2015). Processing strategies and software solutions for data-independent acquisition in mass spectrometry. *Proteomics*.

[13] Wrona M., Mauriala T., Bateman K. P., Mortishire-Smith R. J., O'Connor D. (2005). 'All-in-One' analysis for metabolite identification using liquid chromatography/hybrid quadrupole time-of-flight mass spectrometry with collision energy switching. *Rapid Communications in Mass Spectrometry*.

[14] Bonner R., Hopfgartner G. (2016). SWATH acquisition mode for drug metabolism and metabolomics investigations. *Bioanalysis*.

[15] Gillet L. C., Navarro P., Tate S. (2012). Targeted data extraction of the MS/MS spectra generated by data-independent acquisition: a new concept for consistent and accurate proteome analysis. *Molecular & Cellular Proteomics*.

[16] Exton J. H. (1994). Phosphatidylcholine breakdown and signal transduction. *Biochimica et Biophysica Acta (BBA) - Lipids and Lipid Metabolism*.

[17] Murakami I., Wakasa Y., Yamashita S. (2011). Phytoceramide and sphingoid bases derived from brewer’s yeast saccharomyces pastorianus. *Lipids in Health and Disease*.

[18] Floegel A., Stefan N., ZH Y. (2013). Identification of serum metabolites associated with risk of type 2 diabetes using a targeted metabolomics approach. *Diabetes*.

[19] Muoio D. M. (2012). Revisiting the connection between intramyocellular lipids and insulin resistance: A long and winding road. *Diabetologia*.

[20] Botero-Ruiz W., Laufer N., DeCherney A. H. (1984). The relationship between follicular fluid steroid concentration and successful fertilization of human oocyte in vitro. *Fertility and Sterility*.

[21] Wu L. L., Chiou C.-C., Chang P.-Y., Wu J. T. (2004). Urinary 8-OHdG: a marker of oxidative stress to DNA and a risk factor for cancer, atherosclerosis and diabetics. *Clinica Chimica Acta*.

[22] Foucaud L., Goulaouic S., Bennasroune A. (2010). Oxidative stress induction by nanoparticles in THP-1 cells with 4-HNE production: stress biomarker or oxidative stress signalling molecule?. *Toxicology in Vitro*.

[23] Xu B., Guo N., Zhang X.-M. (2015). Oocyte quality is decreased in women with minimal or mild endometriosis. *Scientific Reports*.

[24] Ben-Meir A., Burstein E., Borrego-Alvarez A. (2015). Coenzyme Q10 restores oocyte mitochondrial function and fertility during reproductive aging. *Aging Cell*.

[25] Feng M. Y., Li G. H., Xu Z. P. (2014). Effects of L-carnitine on oocytes in vitro maturation, lipid metabolism and parthenogenetic embryos development in porcine. *Journal of South China Agricultural University*.

[26] Zare Z., Masteri Farahani R., Salehi M. (2015). Effect of L-carnitine supplementation on maturation and early embryo development of immature mouse oocytes selected by brilliant cresyle blue staining. *Journal of Assisted Reproduction and Genetics*.

